# Calibration of a serum reference standard for Group B streptococcal polysaccharide conjugate vaccine development using surface plasmon resonance

**DOI:** 10.1038/s41541-023-00667-1

**Published:** 2023-05-19

**Authors:** Alexandre Esadze, Christopher D. Grube, Sabine Wellnitz, Suddham Singh, Ha H. Nguyen, Michelle A. Gaylord, Aiping Zhu, Alexey Gribenko, Charles Y. Tan, Annaliesa S. Anderson, Raphael Simon

**Affiliations:** 1 Pfizer Vaccine Research and Development, Pearl River, NY 10965 USA; 2 Pfizer Worldwide Research and Development, Pearl River, NY 10965 USA; 3 Pfizer Worldwide Research and Development, Collegeville, PA 19426 USA

**Keywords:** Conjugate vaccines, Conjugate vaccines

## Abstract

Group B streptococcus (GBS) is a leading cause of neonatal morbidity and mortality worldwide. Development of a maternal vaccine to protect newborns through placentally transferred antibody is considered feasible based on the well-established relationship between anti-GBS capsular polysaccharide (CPS) IgG levels at birth and reduced risk of neonatal invasive GBS. An accurately calibrated serum reference standard that can be used to measure anti-CPS concentrations is critical for estimation of protective antibody levels across serotypes and potential vaccine performance. For this, precise weight-based measurement of anti-CPS IgG in sera is required. Here, we report an improved approach for determining serum anti-CPS IgG levels using surface plasmon resonance with monoclonal antibody standards, coupled with a direct Luminex-based immunoassay. This technique was used to quantify serotype-specific anti-CPS IgG levels in a human serum reference pool derived from subjects immunized with an investigational six-valent GBS glycoconjugate vaccine.

## Introduction

Group B streptococcus (*Streptococcus agalactiae*, GBS) is a Gram-positive, encapsulated pathogen that colonizes the rectovaginal tract of ~15–30% of women worldwide and is a major cause of invasive disease in newborns^1,2^. The capsular polysaccharide (CPS) that envelops the GBS bacterial cell is a key virulence factor and protective antigen in animal models^3–6^. Ten GBS CPS serotypes (Ia, Ib, II–IX) have been identified that are differentiated by the chemical composition and the specific linkage of monosaccharides in the CPS repeat^7–9^. GBS serotypes Ia, Ib, II, III, IV and V account for >98% of disease globally^3^. A vaccine to prevent invasive GBS disease represents an area of unmet medical need and would provide an important public health tool to prevent GBS disease in infants via maternal immunization. Pfizer is developing a six-valent CPS glycoconjugate vaccine (GBS6) that targets the six most prevalent GBS serotypes worldwide through vaccination of pregnant women and placental transfer of anti-CPS antibodies^3,10^.

There are multiple studies for which a general correlation between maternally-derived anti-GBS CPS antibody levels and reduced risk of GBS disease in newborns has been observed with an attempt made to derive an immunological threshold of protection^11–17^. While these studies clearly established that antibodies to GBS capsular polysaccharides in maternal or infant sera are associated with protection against GBS disease in the infants, a consensus protective titer has not been identified, partly because the corresponding studies used different serological assays and associated reference standards.

A common serological assay and associated serum reference standard would permit comparability across studies. Additionally, accurate weight-based characterization of the relative concentrations of anti-CPS antibodies in the reference standard would allow for comparison across serotypes and offer the opportunity to evaluate the feasibility of a universal pan-serotype protective anti-CPS serum antibody concentration that could support an immunological endpoint as the basis for potential licensure of a GBS vaccine^18^.

A 6-plex IgG direct Luminex-based immunoassay (dLIA) was developed to quantitate IgG levels against vaccine-relevant GBS CPS serotypes in human serum^10^. This assay was adopted by an international consortium, and is being used to determine IgG concentrations from clinical and sero-epidemiological studies. In this work, we describe an approach to generate a calibrated reference standard using surface plasmon resonance (SPR) and representative serotype-specific monoclonal antibody standards, which is then coupled with the 6-plex IgG dLIA to accurately and quantitatively determine serum anti-CPS IgG levels. This approach was then used to calibrate a human serum reference pool derived from subjects immunized with an investigational six-valent GBS glycoconjugate vaccine.

## Results

### Determination of anti-CPS Ab concentrations in human serum

The SPR-based anti-CPS Ab quantification method utilizes total IgG extracted from human serum. Accordingly, we first confirmed the robustness and reproducibility of IgG extraction from sera. Through independent extractions using four separate aliquots of the human serum reference pool, the recovery of total IgG was found to be reproducible (Table 2), with the average amount of total IgG extracted being ~9.7 ± 0.1 mg from 1 mL of human serum reference pool. This value is in agreement with estimates of 6–16 mg/mL of total serum IgG in healthy individuals^19^.

**Fig. 1:**
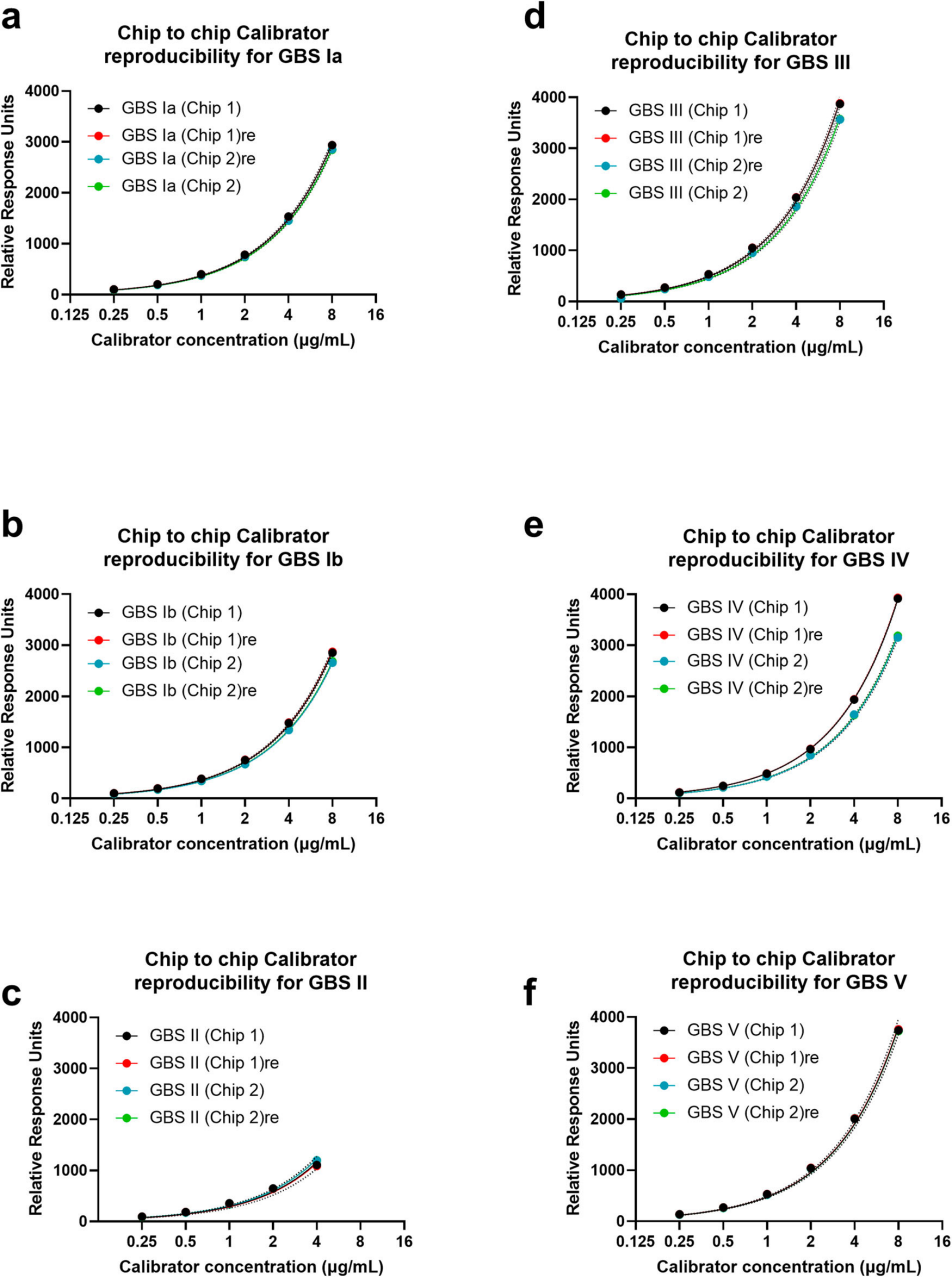
Summary of calibration curves used to validate chip performance. Individual chips were coated with polysaccharide and CPS-PLL conjugates and SPR data were recorded with serotype-specific monoclonal antibodies. **a** GBS Ia CPS, **b** GBS Ib CPS, **c** GBS II CPS, **d** GBS III CPS, **e** GBS IV CPS, **f** GBS V CPS. The “re” suffix denotes that calibration was repeated after running the sample to confirm the chip performance retention. Each panel demonstrates reproducibility of the calibration curves generated for validation of the chip performance. The solid lines represent the fitting done to *Y* = *ax* linear model using the nonlinear regression algorithm of GraphPad Prism. The dotted lines represent the fitting 95% CI.

**Fig. 2:**
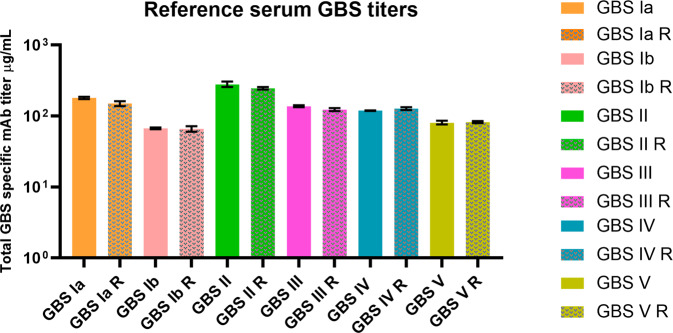
Reproducibility of the method using identically prepared CM5-S-GBS-Poly chips. Polyclonal IgG from a single extraction was tested on separate occasions with two independently prepared chips. Error bars represent two s.d. from the mean. Measurements done on the different chip preparations are plotted side-by-side as solid and checkered bars. To distinguish the two in labeling nomenclature, R is used to denote four repeated replicate measurements done on the second chip. The errors for the solid bars are based on fitting, and for checkered bars, are based on measurements from four biological replicates.

In order to accurately quantitate the amount of anti-CPS antibody present in the purified polyclonal IgG pool, we utilized SPR with calibration to a known amount of a serotype-specific anti-CPS monoclonal IgG antibody (Table 1). For this, binding of GBS serotype-specific IgGs by SPR was measured with four independent polyclonal antibody extractions (Table 3). For each set of sample measurements, calibration curves were run before and after the sample to confirm that the chip performance had not degraded throughout the experiment. We found that the calibration curves overlapped for every serotype (Fig. 1). The potential for variation in GBS serotype-specific levels arising from differences in the immobilized ligand between chip-to-chip preparations was also investigated. For this we assessed the GBS serotype-specific polyclonal IgG pool from a single extraction (replicate #1) reported in Table 2 at two different times, 57 days apart with identically prepared CM5-S-GBS-Poly chips. Figure 1 depicts comparison of calibration curves obtained from the two independently prepared GBS-PLL chips. We found that the calibration curves were reproducible with differences of <5%, with the only exception being GBS serotype IV CPS, where this difference was <13%. Additionally, reproducibility with little variation was seen when comparing IgG levels with two different chips using the same IgG extraction (Table 2 and Fig. 2).

**Table 1:** Overview of antibody clones used in the study.

Clone	Specificity	Fc background
GBS 105-21c	ST Ia	Human IgG1
GBS 17-5c	ST Ib	Human IgG1
GBS 36-9 GBS 328-1c	ST II ST II	Mouse IgG1 Human IgG1
GBS 58-1c	ST III	Human IgG1
GBS 223-5-77c	ST IV	Human IgG1
GBS 67-1c	ST V	Human IgG1

**Table 2:** Reproducibility of total IgG extraction from human serum pool of volunteers vaccinated with GBS6.

Extraction number	Total IgG concentration (µg/mL)	Recovered volume µL	Total IgG isolated µg
1	9600	1000	9600
2	9800	1000	9800
3	9600	1000	9600
4	9100	1055	9600

Chip validation was also performed for each individual serotype. We observed reproducibility of the calibration curves that sandwiched the samples, indicating that the chip performance was unperturbed (surface degradation over time, or post regeneration) during the data acquisition for the samples (Supplementary Fig. 1A–F panel i). This warranted averaging of the two calibration curves (Supplementary Fig. 1A–F panel ii) which was then used for calculation of the antibody concentrations in the sample. Secondly, the presence of a vast excess (50-fold) of nontarget antibodies in the mixture did not hinder the detection of the target antibody (Supplementary Fig. 1A–F panel iii, GBS mixture sample). This is an essential control since the concentration of anti-CPS antibodies is expected to be a fraction of the total IgG concentration in the polyclonal IgG pool extracted from serum. For example, for samples from the immune reference serum, the concentrations of individual GBS-serotype specific Abs were 40–120-fold lower than the total IgG concentration. In most cases, the mAbs were unable to interact with the non-target surface, or the extent of interactions was much lower than that of the surface-specific mAb (e.g., GBSII mAb appeared to have minor cross reactivity with GBSIa-PLL, and the GBSIII mAb appeared to weakly interact with GBSIV-PLL). When the raw signal was converted into a cross reactivity index by normalization to mAb concentrations, this difference became virtually zero (Supplementary Fig. 1A–F panel iv).

**Fig. 3:**
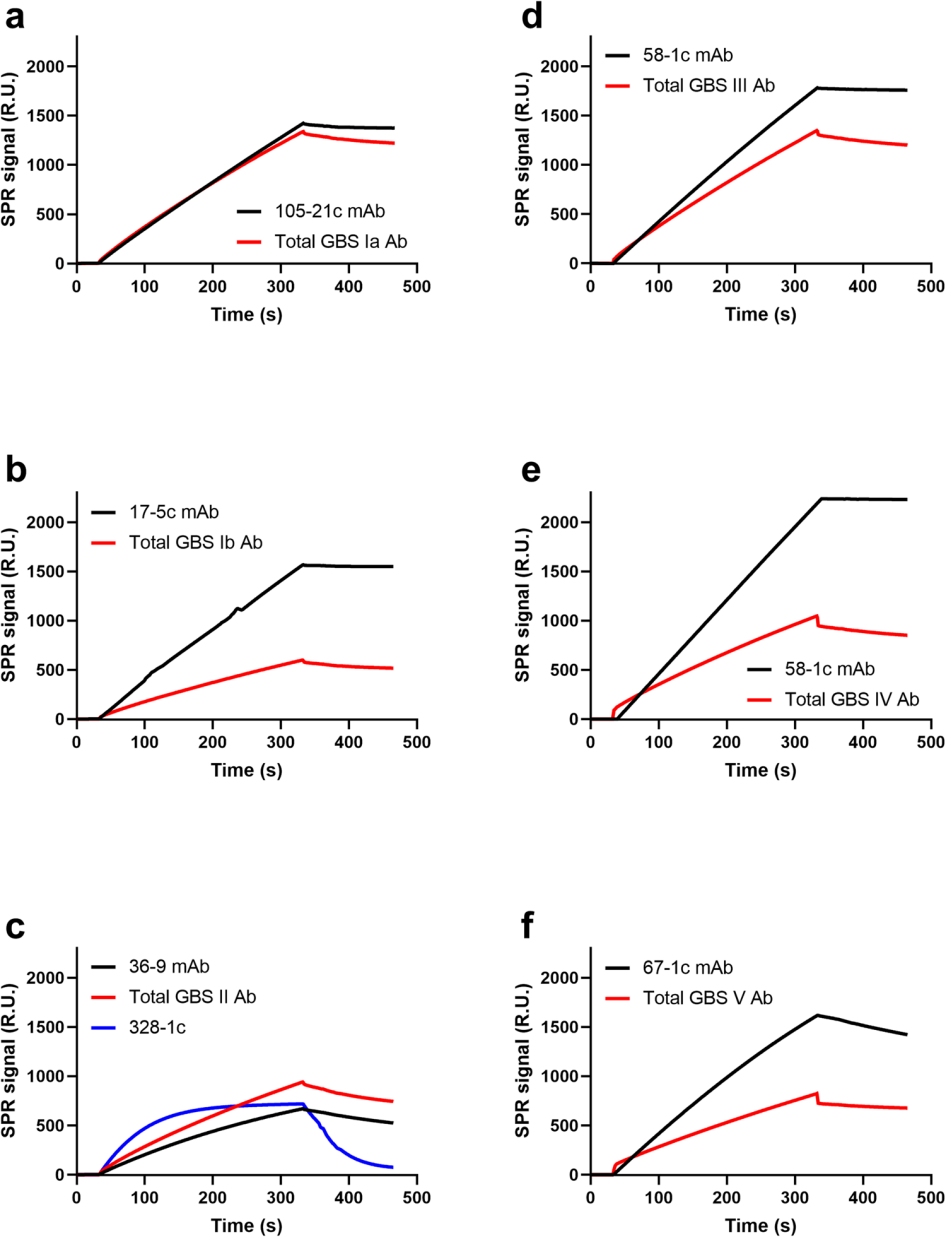
Sensogram traces of anti-CPS antibodies binding to CPS-coated chips. Representative curves depict binding kinetics of serotype-specific mAbs tested at 2 µg/mL along with the relevant total purified polyclonal IgG are shown for: **a** STIa, **b** ST1b, **c** STII, **d** STIII, **e** STIV, **f** STV.

A potential caveat to the use of a single mAb as the calibration reference for a polyclonal Ab mixture is that the interaction pattern of the given mAb may not reflect the apparent interaction pattern arising from the more complex polyclonal antibody mixture. For example, if the dissociation rate of the monoclonal antibody reference is markedly slower than that of the polyclonal serotype-specific IgG, the IgG levels in the serum may be underestimated, as some of the weaker binders may detach and would not contribute to the final signal readout. To exclude systematic errors arising from such a phenomenon, we evaluated the interaction patterns (dissociation) for each of serotype-specific mAbs and the corresponding serotype-specific polyclonal IgG population obtained from human serum (Fig. 3).

An example of obvious divergence between the dissociation rates of calibration mAb and total GBS serotype-specific Abs extracted from human serum is presented in Fig. 3c. Such a calibration mAb, which dissociates much faster than the target sample, would result in significant over-estimation of total serotype-specific IgG levels in the sample. As a result, the calibration mAb (328-1c) was rejected and an alternative was found (mAb 36-9, Fig. 3c) for all subsequent measurements. For the selected calibrator mAbs, we found that the dissociation patterns closely resembled that of the polyclonal antibody population directed against cognate CPS serotype. Having confirmed the robust performance of the method, we then determined the serotype-specific IgG levels in human serum for all six GBS serotypes. We found that the serotype-specific IgG levels had tight distributions and fell within a 95% confidence interval (CI) of the mean (Table 3 and Supplementary Fig. 2).

**Table 3:** GBS serotype-specific IgG level measurements in total IgG extracted from a human serum reference pool composed of subjects vaccinated with GBS6.

	GBS serotype-specific IgG level measurement replicates (µg/mL)			
GBS type	Replicate 1	Replicate 2	Replicate 3	Replicate 4
Ia	156	153	143	146
Ib	68	69	65	62
II	247	252	242	240
III	127	123	123	120
IV	124	131	126	128
V	81	84	81	82

### SPR coupled to dLIA (SPR-dLIA) allows for reproducible, high-throughput measurement of serotype-specific anti-GBS CPS IgG in a human serum reference pool

**Fig. 4:**
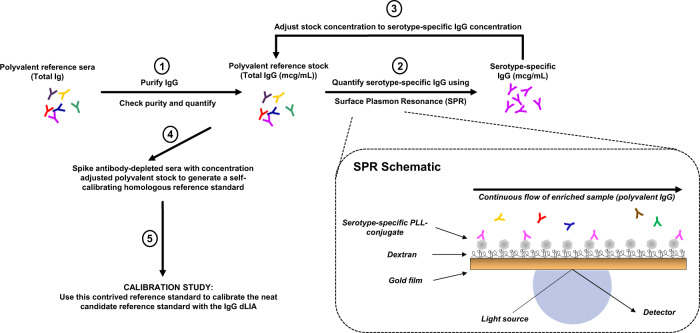
Workflow for the SPR-dLIA method based quantification of GBS serotype-specific anti-CPS IgG in human serum. In steps 1–3, a total IgG stock is isolated from the unmanipulated, polyvalent reference sera, and assigned appropriate serotype-specific IgG level values using the SPR method. In step 4, the purified IgG from the previous steps is used to generate a contrived “self-calibrating” homologous reference standard by spiking antibody-depleted sera (ADS) with the purified IgG. In step 5 of the strategy, the “self-calibrating” homologous reference standard is used as the reference standard in the GBS IgG dLIA, while the unmanipulated, neat reference standard is run as test sample(s) on the assay plates. The reported levels for the neat polyvalent reference standard [run as test sample(s)] from the dLIA are then adjusted based on reference calibration curves from step 5, and accurate weight-based assignments of each serotype-specific IgG are obtained.

**Fig. 5:**
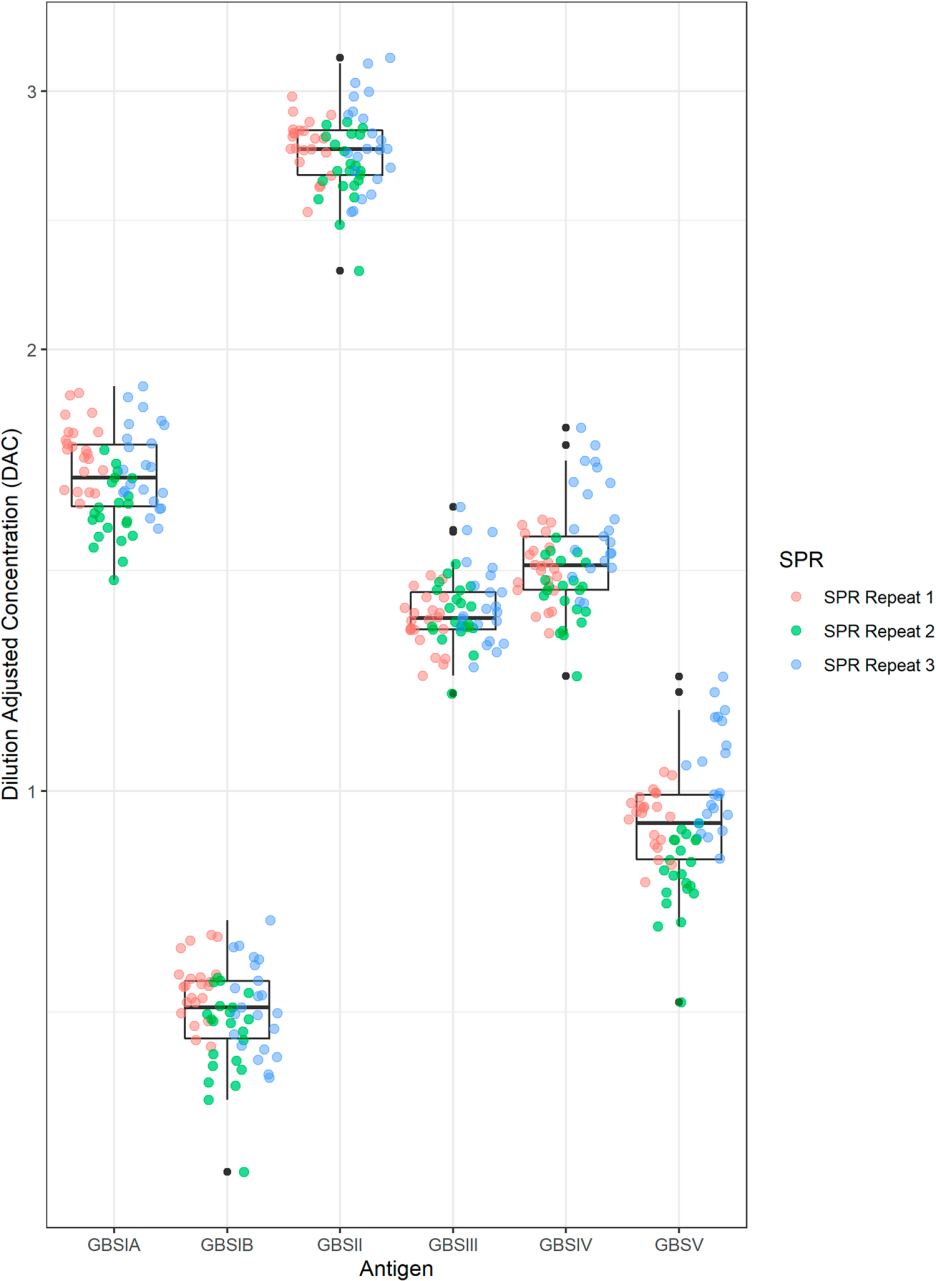
The SPR-dLIA method is highly reproducible across multiple chip preparations and IgG extractions. Each point represents the geometric mean of the three dilution-adjusted concentrations (DAC) of the GBS IGG 2019 material run as test samples in the dLIA. The color codes represent the materials from three individual purifications that were independently quantified via SPR and appropriately diluted in ADS to use as contrived reference standards in the dLIA. Each colored dot represents the paired test samples for those runs. The Y-axis represents the log DAC in µg/mL. Each SPR repeat was run in duplicate (*n* = 22 for each repeat). Points are jittered horizontally to avoid overlapping. The boxes represent 50% of the data around the median. The whiskers at the top and bottom represent ±1.5 IQR (interquartile range). Data points beyond the ±1.5 IQR are represented as black dots.

Following determination of the serotype-specific IgG concentrations in the purified polyclonal IgG pools using SPR, the purified pools were used to calibrate the neat human serum reference pool using the GBS 6-plex dLIA. The workflow schematic for this SPR-dLIA approach is described in Fig. 4. All steps of the workflow were repeated in triplicate to evaluate the reproducibility of the method. In brief, this was accomplished by diluting three purified polyclonal IgG pools in antibody depleted human serum (ADS) in order to generate mock serum samples with known concentrations of anti-CPS IgG for the six GBS serotypes (i.e., homologous reference standard). The “self-calibrating” homologous reference standard was then used as the reference standard in the assay to calibrate the neat human serum reference pool that was run as test sample(s). The assay plate layout for the dLIA can accommodate 11 test samples per run. To take full advantage of the layout, we prepared 11 independent aliquots of the neat human serum reference pool to run as independent test samples. We repeated each dLIA run in duplicate for each condition, resulting in a total of 66 estimates (11 test samples × two dLIA duplicate runs × three homologous reference standards). The results of these experiments are summarized as box-plots in Fig. 5 and indicate that the results are highly reproducible across all repeats and across all trials. The compiled data, along with the 95% CI, are summarized in Table 4.

**Table 4:** SPR-dLIA generated weight-based assignments for the working stock of the human serum reference pool.

GBS serotype	Assignments in mcg/mL (95% Cl^a^)
Ia	1.641 (1.450, 1.858)
Ib	0.709 (0.644, 0.781)
II	2.730 (2.555, 2.915)
III	1.324 (1.271, 1.379)
IV	1.434 (1.251, 1.644)
V	0.952 (0.786, 1.154)

^a^*CI* confidence interval; values from the data source have been rounded for visual representation purposes.

**Fig. 6:**
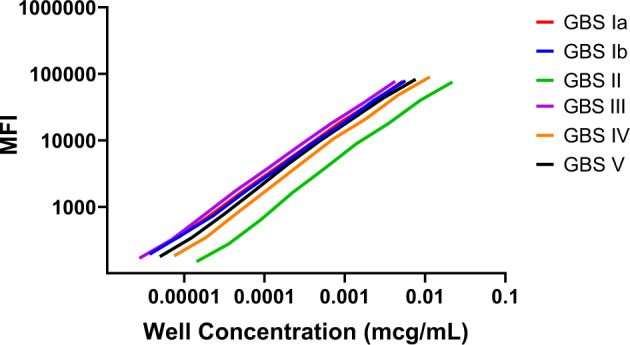
Congruence evaluation of GBS6 serotype, standard curves using the SPR-dLIA generated assignments. Well concentration (*x*-axis) and MFI (*y*-axis) for all six GBS serotypes was plotted for individual serotypes. Data presented are from a single representative experimental run. The 11 individual, serial dilution points were removed from each line for visual clarity.

In addition to assessing the reproducibility of the measurements, the congruence of the standard curves were evaluated by comparing the log-log linear models of the reference curves for all six serotypes to one another using the SPR-dLIA generated assignments. In the evaluation of congruence, the closer the curves are with respect to slope value (i.e., parallelism) and general curve overlay (i.e., observed relationship between sample concentration and MFI values), the stronger the congruence of the serotypes. Based on the results of the congruence assessment, the SPR-dLIA generated values demonstrated strong comparability across five of the six serotypes, the exception being serotype II (Fig. 6).

## Discussion

Seroepidemiological studies have correlated anti-CPS IgG in maternal or infant sera with protection against GBS disease in infants. However, none have used a reference standard that has been cross-standardized to permit comparison of titers between CPS serotypes.

The GBS CPS standard human reference sera (SHRS) developed in the 1990s have been widely adopted by the GBS research community to determine anti-CPS titers in human sera. These sera, derived from volunteers immunized with monovalent CPS vaccines, were assigned weight-based values for anti-IgG CPS antibody indirectly by total antibody immunoprecipitation, and a radioactive antigen binding assay (RABA) followed by correction for the contribution of serotype-specific IgM and IgA by quantitative enzyme-linked immunosorbent assay (ELISA)^20,21^. Though this approach was successful in providing an indication of anti-GBS CPS antibody levels, the way that it was made introduced inherent bias. Methods relying on immunoprecipitation may be subject to error that can arise from incomplete precipitation of antibody-antigen complexes, contribution of other antibody isotypes, and potential variability in the method used to quantitate the precipitated protein. Alternatively, methods based on cross-calibration of ELISA signals from an unknown sample to a parallel plate with a known isotype standard may be influenced by differences in coating conditions and affinities between antigen-specific antibodies and the selected calibrator^22,23^. Importantly, since this method is not cross-standardized across serotypes, comparison of results across serotypes may be imprecise.

Herein, we have detailed a SPR-dLIA approach that overcomes these prior limitations, which can be applied towards weight-based quantitation of CPS serotype-specific IgG in human serum. This method has been developed in order to produce a refined human serum reference standard that could be used as the calibrator for antibody-based assays. Moreover, this method allows for comparison and analysis of IgG concentrations across the six main CPS serotypes associated with the majority of human GBS disease. SPR provides an accurate and readily standardized means to measure antibody binding. Indeed, we documented that the SPR-dLIA method was highly quantitative and reproducible. The use of a monoclonal antibody standard as the calibrator provides an unambiguous weight-based anchor that can be related to the polyclonal antibody sample. The extraction process generates purified IgG and thus permits quantification of serotype-specific total IgG levels in the reference standard without concern for the contribution of other antibody isotypes or diminishing signal effects due to steric interference arising from other serum proteins. Of particular importance for our approach was confirming that the avidity of the representative monoclonal antibodies and the serotype-specific polyclonal IgG in human serum were comparable. The SPR method allows for validation of matched relative avidities of calibrator mAb and relevant investigational IgG mixtures (for example total serotype-specific IgG in the IgG pool purified from serum), which is essential for accurate quantification of specific IgG in complex mixtures. Confirmation of matching avidities provides the basis to disregard any dissociative loss of signal during the SPR measurements, since it would be the same for both standard and sample. Absent such verification, a significant overestimation of the IgG levels could occur, as was demonstrated for reference mAb 328-1c (GBS serotype II). Conventional ELISA and standalone dLIA assays are not capable of such relative avidity comparison, and thus are inherently prone to under- or over-estimation of measured IgG levels due to divergence in relative avidities of target and calibrator.

We found as well that the observed affinity for antibodies specific for serotype II was lower than that seen for antibodies directed against the other GBS CPS serotypes. This may be potentially accounted for by differences in the nature of the polysaccharide structure. Serotype II CPS is an outlier with a short branch of a single sialic acid compared to the other serotypes (Ia, Ib, III, IV, V) that all have trisaccharide branches with terminal sialic acid moieties that may serve as more robust epitopes for antibody binding^8,9^.

The congruence in the MFI as a function of assigned antibody concentration between the reference curves for all six serotypes provided further confirmation for the cross-serotype relatability. Congruence may be expected in this type of assay for two main reasons: (i) given that the CPSs are structurally similar, they should elicit antibody binding profiles with similar affinity and dissociation, and (ii) signal output should be independent of primary antibody-antigen complex formation (result of PE-conjugated secondary Ab) and should be purely representative of weight-based IgG measurements. In the congruence assessment, we observed strong comparability across the serotypes except for GBS II. This can be explained by the observation that the dissociation rate for GBS II from its specific CPS was much faster than for the other serotypes. For standard immunoassay experiments that involve washing steps (i.e., Luminex, ELISA, etc.), a loss of antibodies takes place throughout the course of the assay. This may be reflected in an overall MFI drop at the terminal measurement step, and this loss may explain the GBS II curve’s shift to the right, and lower congruence when compared to the other serotypes’ curves. The characterization and concentration normalization with SPR ensures that all CPS-specific IgG are accounted for when generating the weight-based assignments and highlights a major benefit compared to legacy approaches.

The relative proportion of invasive GBS infections varies across serotypes, with type III and Ia the most prevalent. Accordingly, evaluation of individual protective thresholds in seroepidemiology studies has been feasible for these two serotypes, however, comparable analyses for the minor serotypes has been impeded by their relative infrequency. Definition of a universal protective antibody relationship could be beneficial for licensure of a multivalent GBS CPS vaccine based on an immunological endpoint that would include both major and minor GBS CPS serotypes^24^. Such an approach has precedent as the basis of licensure for newer generations of pneumococcal conjugate vaccines, where a single protective threshold for anti-CPS IgG has been applied across serotypes. The SPR-dLIA technique presented here provides an accurate method for determining GBS serotype-specific IgG concentrations in sera that improves upon legacy approaches. Since this method permits comparison of IgG levels across serotypes, calibration of a serum reference standard with this approach could enable the elaboration and confirmation of a universal cross-serotype protective threshold when used to analyze antibody concentrations from natural history studies, thus enabling the identification of a common set of protective thresholds that could be applied to all serotypes.

As the approach may be generally applicable to measurement of anti-polysaccharide antibodies, it could also be used to calibrate antibody reference standards for polysaccharide antigens derived from other pathogens. This could include calibration of serum reference standards for *Streptococcus pneumoniae*, where circulating antibody concentrations have been related to a defined protective threshold for anti-capsule polysaccharide IgG levels, and legacy reference standards were established by less precise methods^25–27^. An additional application may be to quantify serum antibody levels for the O-antigen of lipopolysaccharide that is the surface polysaccharide for other medically important gram-negative bacteria such as non-typhoidal *Salmonella*, *Pseudomonas aeruginosa*, or *Vibrio cholerae*^28,29^. This method may also be used to relate antibody titers across different biological matrices representing other physiologically relevant compartments.

## Methods

### Monoclonal antibody reference reagents

Serotype-specific GBS monoclonal antibodies were generated as hybridomas from mice immunized with GBS CPS conjugates. Candidate clones were characterized by enzyme linked immunosorbent assay (ELISA) with GBS CPS antigens. Select hybridoma clones (all IgG1 except for GBS 328-1, an IgG2a; Table 1) were engineered as chimeras (indicated as “c” in clone ID) by replacing the mouse heavy and light chain constant regions with the equivalent sequence of the human constant region. A summary of the monoclonal antibody clones used for this study is provided in Table 1.

### IgG quantification

Quantification of purified reference monoclonal antibodies (mAb) and purified total polyclonal IgG antibodies (Ab) extracted from human sera was accomplished via UV absorbance spectrophotometry. Briefly, an undiluted total IgG antibody pool from human serum or mAbs was loaded onto a Nanodrop-8000 (ThermoFisher) system. Single-channel readout configuration was selected with 1× PBS pH 7.4 as a blank. The UV spectra of the samples were then recorded in 220–350 nm range. After manual correction of spectra for scattering at 320 nm, the corrected absorbance at 280 nm was converted into units of concentration using a universal average mass extinction coefficient for antibodies of *Ɛ*_mAb_ = 1.4 (mg mL)^−1^ cm^−1^^30,31^.

### Preparation of GBS CPS Poly-L-Lysine (PLL)

GBS CPS (Ia, Ib, II, III, IV, and V) conjugates with PLL were generated using 1-cyano-4-dimethylaminopyridinium tetrafluoroborate (CDAP) conjugation chemistry. Briefly, GBS CPS was first buffer exchanged to deionized water using Vivaspin 20 centrifugal filters (VS2051, Sartorius). The buffer-exchanged GBS CPS was then aliquoted into 100 mg portions and lyophilized. For the conjugation reaction, GBS CPS was dissolved in deionized water (20 mL total) in a glass Duran bottle. One mL of freshly prepared CDAP (Sigma) solution (100 mg/mL in acetonitrile) was added to the bottle and stirred at room temperature for 30 s. To the CDAP-activated GBS CPS, 4 mL of 0.2 M aqueous triethylamine was then added and stirred for 2 min at room temperature. Seventy-five mL of 0.2 M sodium bicarbonate buffer (pH 8.8) was then added and rapidly mixed, and then 10 mL of PLL (Sigma) solution (1.6 mg/mL in 0.2 M sodium bicarbonate buffer, pH 8.8) was immediately added. The reaction was then mixed at 150 rpm for 20 h at 4 °C. To quench the conjugation reaction 2 M Glycine (2 mL, prepared in 0.2 M sodium bicarbonate buffer, pH 8.8) was added and the reaction was mixed at 150 rpm for another 2 h at 4 °C followed by stirring at 150 rpm for 1 h at room temperature. The unconjugated material was removed by diafiltration using a 100,000 MWCO PES Pellicon XL membrane. For this, the reaction was first concentrated and then diafiltered against 10 diavolumes of 1× PBS + 1 M NaCl buffer, then passed through a 0.22-μm filter (Millipore) and stored at 5 ± 3 °C. The total saccharide content was characterized via Anthrone assay^32^, the poly-lysine content was characterized by 2,4,6-trinitrobenzene sulfonic acid (TNBS) assay^33^ and free CPS content was analyzed via a fractogel approach^34^. The efficiency of the PLL conjugation was found to be >80%. The typical purity of the final product was >90%.

### CM5-S-GBS-Poly chip preparation and conditioning for detection of GBS serotype-specific antibodies

To generate custom CM5-S-GBS-Poly chips, poly-L-lysine-conjugated GBS CPS was crosslinked with the blank CM5-S (Cytiva) chip using an Amine coupling kit (Cytiva) and the manufacturer’s recommended procedures. A sodium acetate pH 4.0 condition was used for all crosslinking reactions. The concentration of GBS CPS-PLL conjugates used in the crosslinking is shown in Supplementary Table 1. The total contact time of GBS CPS-PLL conjugates with the CM5-S surface was 300 seconds at application flow rate of 10 µL/min. All the other conditions were as suggested by the crosslinking kit manufacturer. Sample and reagents were loaded on racks in accordance with the Biacore T-200 auto generated sample map. After crosslinking, the chips were washed, equilibrated in neutral pH, and verified before the quantitation of GBS serotype-specific IgG levels (Supplementary Fig. 1). For quantitation, 8, 4, 2, 1, 0.5, 0.25 or 0 (buffer alone) µg/mL of reference GBS serotype-specific mAb was applied to the chip to generate a standard curve. The standard curve was followed by a mock sample of 1 µg/mL of the same reference mAb. Next, relevant negative control reference mAbs (not specific to the GBS CPS) were injected at 10 µg/mL to determine the degree of non-specific interaction. Finally, a mixture of 1 µg/mL of specific mAb and 10 µg/mL of each non-specific mAb was applied as a negative control to determine if an excess of non-specific antibodies hinders the quantification of specific antibody. The standard curve injections were then repeated to verify the performance of the chip post-sample loading. The running buffer for all experiments was 1× HBS-P (Hepes buffered saline pH 7.4 containing P20 surfactant prepared from 20× HBS-P, TEKNOVA). Three cycles were conducted for surface conditioning and startup with 1× HBS-P buffer for all experiments. The sample/reference contact time was 180 s at 10 µL/min loading speed. A 60-second stabilization was then allowed before signal readout. Regeneration was performed after each injection with nearly saturated MgCl_2_ (filtered through 0.45 µm) for 120 s at a 50 µL/min rate followed by a second regeneration with 10 mM Glycine-HCl pH 1.5 (part of regeneration scouting kit, Cytiva, BR-1005-56) for 120 seconds at 50 µL/min. The experimental setup of reagent and sample loading was done in accordance with Biacore T200 autogenerated map.

### Verification of the CM5-S-GBS-Poly chip surfaces

To verify the quality of the produced CM5-S-GBS-Poly chips, each coated with distinct GBS-PLL, we assessed: (1) the reproducibility of calibration curves generated by the reference standard mAb specific to the chip surface; (2) cross-reactivity index, or the fractional extent to which mAbs specific to other serotypes (but not to the CPS to which it was applied) interacted with the particular CM5-S-GBS-PLL surface; and (3) the ability of the chip surfaces to detect target mAb in the mixture with an excess of non-target antibodies over the target mAb (see main text and Supplementary Fig. 1).

### Extraction of total IgG from human reference serum pool

One mL aliquots of Pfizer’s human serum reference pool, derived from subjects immunized with an investigational six-valent GBS glycoconjugate vaccine (NCT03170609), were stored at −80 °C until use. Serum aliquots were thawed at room temperature, carefully mixed and then spun down in a tabletop Eppendorf centrifuge for 30 s at 4000 × *g*. A 3.75 mL volume of Magne^TM^ protein A (Promega) and 3.75 mL of Magne^TM^ protein G (Promega) magnetic bead slurries were combined in a 15 mL centrifuge tube (Falcon). The tube was then placed in a magnetic separator (Promega) and the storage buffer was carefully aspirated. The bead mixture was then washed five times with 1× PBS pH 7.4 (Corning). The 1 mL serum aliquot was diluted with 6 mL of 1× PBS (pH 7.4) to a final volume of 7 mL and added to the 1.5 mL of dry protein A/G magnetic bead mixture. The Falcon tube was then capped and sealed with parafilm, and incubated for approximately 12 h at 4 °C with gentle end-over-top mixing in a rotary mixer (SCI Logex). The tubes were then placed in a magnetic separator and the depleted, diluted IgG serum was aspirated. Beads were then washed with 8 mL of 1× PBS, pH 7.4 five times. Elution of IgG was performed by washing magnetic beads with 5 mL of 30 mM sodium acetate pH 2.8 for 1 min at room temperature with gentle agitation. The tubes were then set in a magnetic separator, the eluent containing IgG was aspirated and immediately neutralized with 1.2 mL of 1 M Tris-HCl pH 8.0 (Corning). The elution was repeated five times to ensure complete recovery of the captured IgG. The optical density (OD) at 280 nm for each elution step was measured on a Nanodrop-8000 as described above. The eluted IgG from the first four elution steps was pooled together and immediately buffer-exchanged to 1× PBS, pH 7.4 with at least a 10,000-fold dilution factor of the original buffer contents using 10 K MWCO Amicon® Ultra-15 centrifugal filters (MilliporeSigma), centrifuged at 4000 × *g*. Finally, total IgG from each extraction was concentrated to ~500 µL and diluted to 1000 µL (original serum aliquot volume) with 1× PBS pH 7.4, and stored at 4 °C.

### Determination of GBS CPS serotype-specific IgG levels and evaluation of the reproducibility of GBS serotype-specific Ab concentration

For the measurement of GBS CPS serotype-specific polyclonal antibody levels in the purified total IgG pool from human serum, extracted total IgG was freshly diluted 1:90 in 1× HBS-P to prevent overloading of the chip. The raw SPR signal from the target samples were corrected based on dilution factor and background signals. The corrected value of the sample signal was then converted into concentration using the linear dependence between the SPR signal and concentration for the reference curve. The calibration curves for quantification of the serotype-specific total Abs were measured pre- and post-sample loading. After correction of the calibration curves to the background contributions, the results were averaged and plotted on a log-linear plot using GraphPad Prism software. The calibration curves were fitted to a simple linear equation that crosses the point of origin using a nonlinear regression routine of GraphPad Prism:

1 $${\rm{R}}.{\rm{U}}.\,=\,{\alpha}{x}$$

Where R.U. is SPR signal measured in resonance units by Biacore, *x* is the concentration in µg/mL plotted on the *x*-axis, and *α* is the coefficient that connects the SPR signal in resonance units with the known concentration of the calibration or test sample in µg/mL. The GBS serotype-specific Ab concentrations in the test sample were then calculated via the following equation:

2 $${C}_{{{\rm{sample}}}}=\frac{\left({{\rm{R}}.{\rm{U}}.}_{{{\rm{sample}}}}\,-\,{{\rm{R}}.{\rm{U}}.}_{{{\rm{zero}}}}\right){{\cdot}}\,{\rm{dilution}}\; {\rm{factor}}}{\alpha}$$

*C*_sample_ is the concentration of GBS serotype-specific total IgG in the tested sample in µg/mL, R.U._sample_ is the SPR signal measured by Biacore for the sample, R.U._zero_ is the signal measured for the 0 µg/mL point for the reference curve standards, and dilution factor is the dilution level of the sample loaded onto the chip and in all cases is equal to 90.

The assessment of reproducibility of GBS serotype-specific level measurements was performed by measuring specific Ab levels in four independent IgG extractions calculating the average values of the levels and standard deviations (s.d.), and then evaluating the distribution of the measured values within two s.d. (95% CI) of the average value calculated. To assess the variations in GBS serotype-specific Ab level measurements introduced via different chip preparation, GBS serotype-specific Ab levels measured on identically prepared and verified CM5-S-GBS-Poly chips 57 days apart were compared. The average of the measurements and s.d. were calculated via the following equations:

3 $${{\rm{Average}}}=\frac{\sum {{\rm{Replicate}}}\;{{\rm{measurements}}}}{N}$$

4 $${{\rm{Standard}}}\;{{\rm{deviation}}}=\,\sqrt{\mathop{\sum }\limits_{1}^{N}\frac{{({x}_{i}-{\rm{\mu }})}^{2}}{N-1}}$$

Here *x*_*i*_ is the *i*th measurement made, *µ* is the average value calculated via Eq. 3, and *N* is the total number of measurements made. Summation is done over all *N* values measured.

### Determination of serotype-specific GBS IgG concentrations in a human serum reference pool using a 6-plex direct Luminex immunoassay (dLIA)

We previously reported the development of a 6-plex GBS dLIA to measure GBS polysaccharide-specific (serotypes Ia, Ib, II, III, IV, V) IgG antibodies in human serum samples using six sets of spectrally distinct magnetic polystyrene microspheres coupled to GBS CPS-PLL conjugates^10^. Briefly, for the primary incubation step, 50 μL of GBS CPS-PLL-coupled microspheres (5 × 10^4^ microspheres/mL per serotype) were incubated overnight with shaking (MaxQ 2000 shaker, 300 RPM) at 4 °C with 50 μL of human serum reference pool, quality control samples (QCS), and test serum samples that are run in duplicate and have been appropriately diluted in assay buffer (0.5% BSA in 10 mM PBS/0.05% Tween-20/0.02% Sodium Azide, pH 7.2) in 96-well microtiter plates (Costar Cat. #3912). The following day, the assay plates underwent three wash cycles using 100 μL of wash buffer (7.8 mM Na_2_HPO_4_•7H_2_O/2.2 mM KH_2_PO_4_/0.137 M NaCl/2 mM KCl, 0.02% NaN_3_, 0.05% Tween-20, [Northeast Laboratories]) with a Tecan HydroSpeed™ plate washer (magnetic bead attachment) to remove non-bound components. Following the wash step, 50 μL of a R-Phycoerythrin-conjugated goat anti-human IgG secondary antibody (Jackson ImmunoResearch Cat. #109-115-098) that had been diluted 1:500 in assay buffer, was added to the wells of the plate for a 90 ± 30-min secondary incubation step performed at room temperature with shaking (MaxQ 2000 shaker, 300 RPM). PE was used to detect and quantify specific anti-CPS IgG antibodies bound to the beads. The assay plates were read on a Luminex^®^ reader (e.g., FLEXMAP 3D^®^) after the final wash and resuspension steps (100 μL of wash buffer) were performed. The signal output is expressed as median fluorescent intensities (MFIs), which are evaluated against the human serum reference pool with weight-based IgG assignments (in µg/mL) for each serotype. Raw data were converted to IgG levels using a log–log linear regression model in Pfizer’s validated Statistical Analysis System (SAS^®^).

### Reporting summary

Further information on research design is available in the Nature Research Reporting Summary linked to this article.

## Supplementary information

Supplementary Materials

Reporting Summary

## Data Availability

All output raw and processed data files are available upon reasonable request to corresponding author.
