# Supplementary material for: Calibration of a serum reference standard for Group B streptococcal polysaccharide conjugate vaccine development using surface plasmon resonance
Source: NPJ Vaccines. 2023 May 19;8:71. doi: 10.1038/s41541-023-00667-1 (PMC10197048; doi:10.1038/s41541-023-00667-1)
Supplement: Supplementary file 1 — Supplementary Materials [file 41541_2023_667_MOESM1_ESM.pdf]

SUPPLEMENTARY INFORMATION

Supplementary Tables

Supplementary Table 1. GBS CPS-PLL conjugate concentrations[1] used to prepare CM5 S-GBS-poly chips.

GBS PLL	Concentration used (µg/mL)
GBS Ia PLL	20
GBS Ib PLL	15
GBS II PLL	10
GBS III PLL	15
GBS IV PLL	6
GBS V PLL	6

[1]The optimal concentrations of the GBS poly-Lys (GBS PLL) conjugates used in amine cross slinking immobilization to CM5-S chip dextran surface. The optimal condition reflects the amount of the GBS PLL that gave maximal signal in pre-concentration scouting experiments, thus providing the maximal GBS PLL deposition on the dextran matrix for subsequent crosslinking.

Supplementary Figures

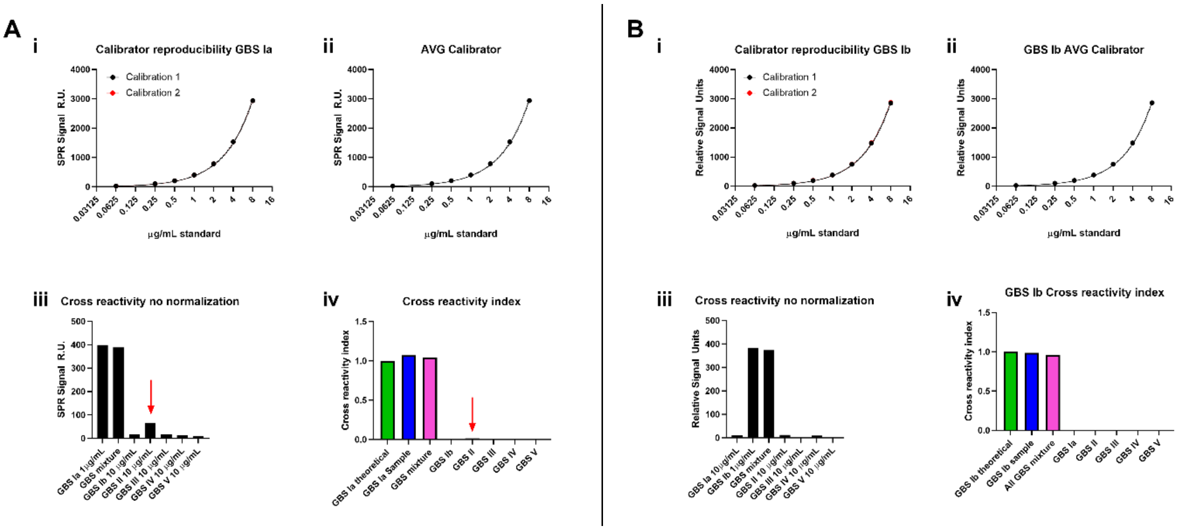

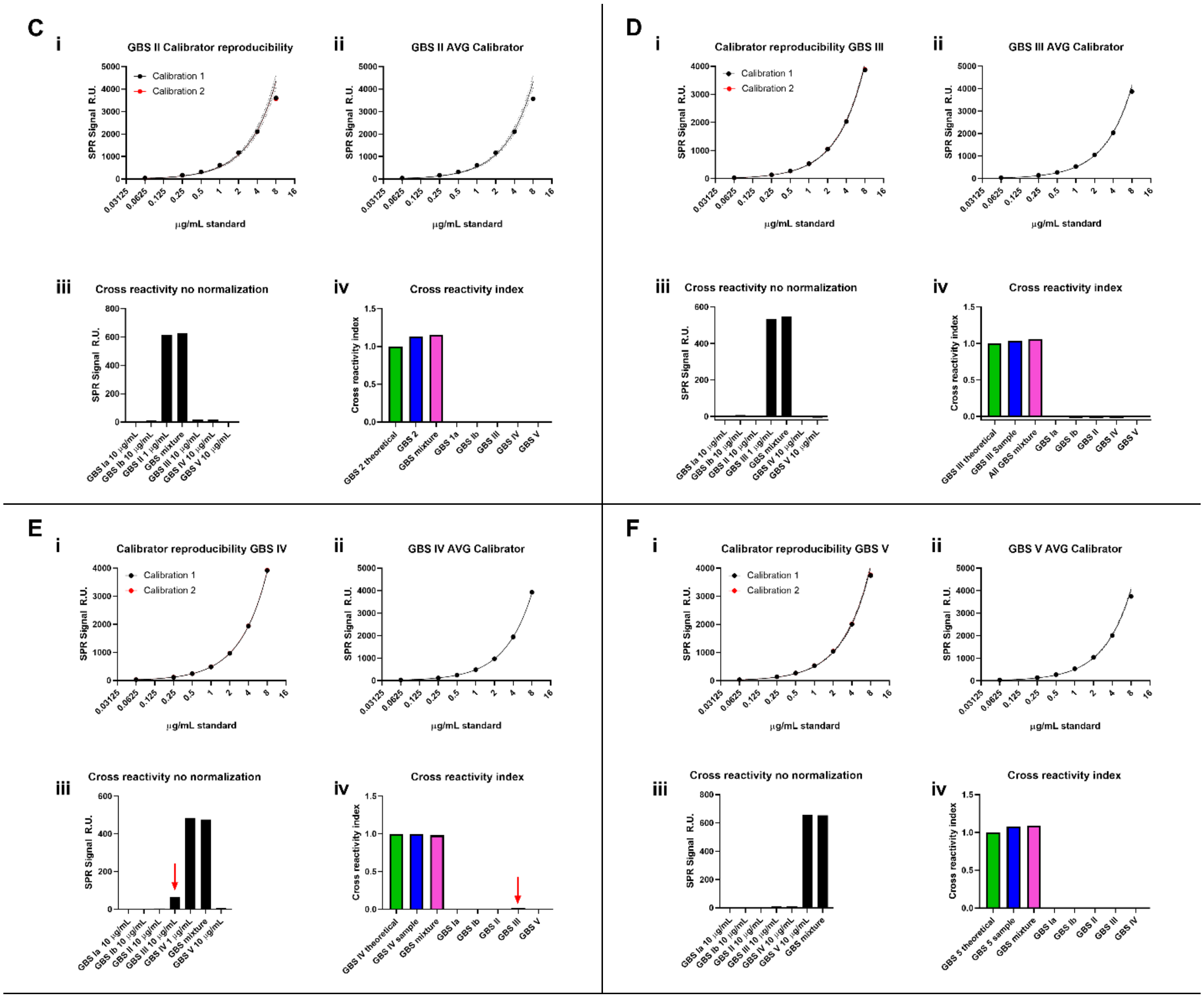

Supplementary Figure 1. Monoclonal reference antibodies yield highly specific and reproducible calibration curves. Calibrator antibodies for CPS serotypes Ia (A), Ib (B), II (C), III (D), IV (E) and V (F) were assessed by SPR with the homologous serotype shown individually (panel i) and as an average of the two replicates (panel ii). Cross-reactivity was also assessed with individual mAbs and a mixture of all 6 mAbs with the homologous and heterologous CPS serotype (iii). GBS mixture samples contained 10 µg/mL of each 5 non-target mAbs and 1 µg/mL of the target mAb. The cross-reactivity index (panel iv) represented the concentration-normalized SPR signals from panel iii. An index value of 1 indicated that specific and nonspecific antibodies were indistinguishable, whereas an index value of 0 indicated nonspecific interaction was completely absent.

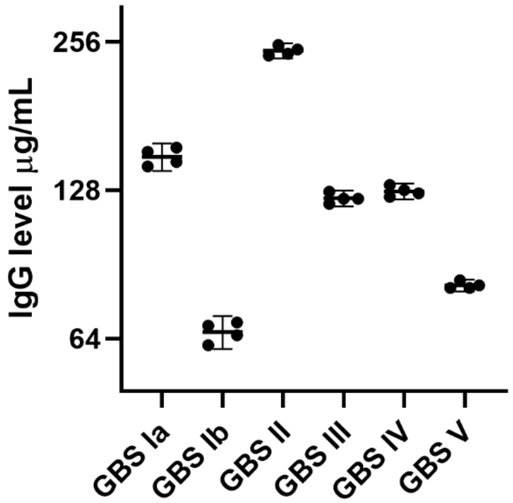

Supplementary Figure 2. The SPR-based assay provides for reproducible and accurate measurements of serotype-specific IgG levels in human serum. Data from four independent replicates are shown for each serotype, with the average and 95% confidence interval (CI) shown.
