# Supplementary material for: Calibration of a serum reference standard for Group B streptococcal polysaccharide conjugate vaccine development using surface plasmon resonance
Source: NPJ Vaccines. 2023 May 19;8:71. doi: 10.1038/s41541-023-00667-1 (PMC10197048; doi:10.1038/s41541-023-00667-1)
Supplement: Supplementary file 2 — Reporting Summary [file 41541_2023_667_MOESM2_ESM.pdf]

nature portfolio

Corresponding author(s): Raphael Simon

Last updated by author(s): Jan 26, 2023

Reporting Summary

Nature Portfolio wishes to improve the reproducibility of the work that we publish. This form provides structure for consistency and transparency in reporting. For further information on Nature Portfolio policies, see our Editorial Policies and the Editorial Policy Checklist.

Statistics

For all statistical analyses, confirm that the following items are present in the figure legend, table legend, main text, or Methods section.

n/a	Confirmed	
☐	☒	The exact sample size (n) for each experimental group/condition, given as a discrete number and unit of measurement
☐	☒	A statement on whether measurements were taken from distinct samples or whether the same sample was measured repeatedly
☒	☐	The statistical test(s) used AND whether they are one- or two-sided<br>*Only common tests should be described solely by name; describe more complex techniques in the Methods section.*
☒	☐	A description of all covariates tested
☒	☐	A description of any assumptions or corrections, such as tests of normality and adjustment for multiple comparisons
☐	☒	A full description of the statistical parameters including central tendency (e.g. means) or other basic estimates (e.g. regression coefficient) AND variation (e.g. standard deviation) or associated estimates of uncertainty (e.g. confidence intervals)
☒	☐	For null hypothesis testing, the test statistic (e.g. F, t, r) with confidence intervals, effect sizes, degrees of freedom and P value noted<br>*Give P values as exact values whenever suitable.*
☒	☐	For Bayesian analysis, information on the choice of priors and Markov chain Monte Carlo settings
☒	☐	For hierarchical and complex designs, identification of the appropriate level for tests and full reporting of outcomes
☒	☐	Estimates of effect sizes (e.g. Cohen's d, Pearson's r), indicating how they were calculated

Our web collection on statistics for biologists contains articles on many of the points above.

Software and code

Policy information about availability of computer code

Data collection	No custom code was used for data analysis or collection. Raw data were analyzed in Pfizer's validated Statistical Analysis System (SAS®) and GraphPad Prism.
Data analysis	No custom code was used for data analysis or collection. Raw data were analyzed in Pfizer's validated Statistical Analysis System (SAS®) and GraphPad Prism.

For manuscripts utilizing custom algorithms or software that are central to the research but not yet described in published literature, software must be made available to editors and reviewers. We strongly encourage code deposition in a community repository (e.g. GitHub). See the Nature Portfolio guidelines for submitting code & software for further information.

Data

Policy information about availability of data

All manuscripts must include a data availability statement. This statement should provide the following information, where applicable:

- Accession codes, unique identifiers, or web links for publicly available datasets
- A description of any restrictions on data availability
- For clinical datasets or third party data, please ensure that the statement adheres to our policy

All output raw and processed data files are available upon reasonable request to corresponding author.

Human research participants

Policy information about studies involving human research participants and Sex and Gender in Research.

Reporting on sex and gender	N/A
Population characteristics	N/A
Recruitment	N/A
Ethics oversight	N/A

Note that full information on the approval of the study protocol must also be provided in the manuscript.

Field-specific reporting

Please select the one below that is the best fit for your research. If you are not sure, read the appropriate sections before making your selection.

☒ Life sciences ☐ Behavioural & social sciences ☐ Ecological, evolutionary & environmental sciences

For a reference copy of the document with all sections, see nature.com/documents/nr-reporting-summary-flat.pdf

Life sciences study design

All studies must disclose on these points even when the disclosure is negative.

Sample size	Different sample (pooled IgG from human sera) batches were tested for an initial evaluation of method precision and accuracy.
Data exclusions	No data were excluded.
Replication	The data were consistent between assays and can be replicated. Experiments were performed once unless otherwise stated.
Randomization	The trial was randomized and placebo-controlled, however, for the purpose of this study, only experimental samples (sera from vaccinated subjects) were used.
Blinding	The trial was performed in a a blinded fashion. For the purpose of this study, researchers were only interested in the experimental group (vaccinated subjects) and therefore, samples were unblinded prior to testing.

Reporting for specific materials, systems and methods

We require information from authors about some types of materials, experimental systems and methods used in many studies. Here, indicate whether each material, system or method listed is relevant to your study. If you are not sure if a list item applies to your research, read the appropriate section before selecting a response.

Materials & experimental systems

n/a	Involved in the study	
☐	☒	Antibodies
☒	☐	Eukaryotic cell lines
☒	☐	Palaeontology and archaeology
☒	☐	Animals and other organisms
☐	☒	Clinical data
☒	☐	Dual use research of concern

Methods

n/a	Involved in the study	
☒	☐	ChIP-seq
☒	☐	Flow cytometry
☒	☐	MRI-based neuroimaging

Antibodies

Antibodies used	All monoclonal antibodies were generated by our in-house reagent team for our group b streptococcal vaccine program. Clone names: GBS 105-21c, GBS-17-5c, GBS 36-9, GBS 328-1c, GBS 58-1c, GBS 223-5-77c, GBS 67-1c. A R-Phycoerythrin-conjugated goat anti-human IgG (Jackson ImmunoResearch, #109-115-098, polyclonal) was used as a secondary antibody in the dLIA.
Validation	All monoclonal antibodies, and their application in this study, were validated in our in-house group b streptococcal vaccine program.

Clinical data

Policy information about clinical studies

All manuscripts should comply with the ICMJE guidelines for publication of clinical research and a completed CONSORT checklist must be included with all submissions.

Clinical trial registration	NCT03170609
Study protocol	Full trial protocol can be accessed at: https://clinicaltrials.gov/ProvidedDocs/09/NCT03170609/Prot_000.pdf
Data collection	Study began on June 5, 2017 and completed on June 25, 2018. Samples were collected from three U.S. cities: Stockbridge, GA; Bardstown, KY; Salt Lake City, UT. Study participants were adults (all sexes) ages 18-49.
Outcomes	Details on assessment of trial outcomes can be accessed at: https://clinicaltrials.gov/ProvidedDocs/09/NCT03170609/SAP_001.pdf
